# Combined effects of biochar and chicken manure on maize (*Zea mays* L.) growth, lead uptake and soil enzyme activities under lead stress

**DOI:** 10.7717/peerj.11754

**Published:** 2021-07-12

**Authors:** Ling Liu, Jiwei Li, Guanghai Wu, Hongtao Shen, Guozhan Fu, Yanfang Wang

**Affiliations:** 1 College of Agriculture, Henan University of Science and Technology, Luoyang, Henan, China; 2 State Key Laboratory of Soil Erosion and Dryland Farming on the Loess Plateau, Northwest Agriculture and Forestry University, Yangling, Shaanxi, China; 3 China Tobacco Henan Industrial Limited Company, Zhengzhou, Henan, China

**Keywords:** Lead uptake, Biochar, Chicken manure, Maize growth, Antioxidant enzymatic activities, Bioconcentration factor

## Abstract

The goal of the present work was to evaluate the additive effects of biochar and chicken manure on maize growth in Pb-contaminated soils. In this study, we conducted a pot experiment to investigate how biochar in soil (20, 40 g·kg^−1^), chicken manure in soil (20, 40 g·kg^−1^), or a combination of biochar and chicken manure in soil (each at 20 g·kg^−1^) effect maize growth, Pb uptake, leaves’ antioxidant enzymatic activities, and soil enzyme activities under artificial conditions to simulate moderate soil pollution (800 Pb mg·kg^−1^). The results showed that all biochar and/or chicken manure treatments significantly (*P* < 0.05) increased maize plant height, biomass, and superoxide dismutase (SOD), peroxidase (POD), and catalase (CAT) activity but decreased the malondialdehyde (MDA) content. These results indicated that amending the soil with biochar and/or chicken manure could alleviate Pb’s phytotoxicity. The biochar and/or chicken manure treatments remarkably decreased the Pb concentration in maize roots, stems, leaves, bioconcentration factor (BCF), translocation factor (TF), and available Pb concentration in the soil. Amending the soil with chicken manure alone was more effective at increasing maize growth and antioxidant enzymatic activity; the biochar treatment alone was more effective at inducing soil alkalinization and contributing to Pb immobilization. The combined use of biochar and chicken manure had an additive effect and produced the largest increases in maize growth, leaves’ antioxidant enzymatic activity, and soil enzyme activity. Their combined use also led to the most significant decreases in maize tissues Pb and soil available Pb. These results suggest that a combination of biochar and chicken manure was more effective at reducing soil Pb bioavailability and uptake by maize tissues, and increasing maize growth. This combination increased plant height by 43.23% and dry weight by 69.63% compared to the control.

## Introduction

Recently, soil contamination of agricultural land throughout the world by heavy metals has become a serious problem. Heavy metals are toxic for plants and can inhibit plant growth, development, and productivity (Chernyshuk et al., 2020; Zhang et al., 2018). Lead (Pb) is a classically deleterious heavy metal that threatens agro-ecosystem sustainability through anthropogenic activities such as mining, waste disposal, and the intensive use of pesticides and fertilizers (Adler et al., 2016; Fu & Wang, 2011). Pb is easily accumulated in soil and is readily absorbed by plants, which may cause harm to human health through the food chain (Deng et al., 2014; Ma et al., 2016). Therefore, the remediation of Pb contaminated soil is critical to ensure soil security and the sustainable development of agriculture (García-Delgado et al., 2019). Modern remediation approaches, including physical extraction, chemical immobilization, and bioremediation have been used to alleviate heavy metal toxicity in soils and improve plant performance (Diaconu et al., 2020; Hu et al., 2019; Meng et al., 2020). Previous studies have shown that organic soil amendments may immobilize heavy metals, and are regarded as an environmentally friendly and economically feasible process (Jiang et al., 2012; Lahori et al., 2017; Zama et al., 2018). Adding organic amendments may change heavy metal speciation by precipitation, adsorption, and ion exchange reactions (Li et al., 2017; Liu et al., 2012). Organic amendments may also decrease heavy metal concentrations in plants and available heavy metal contents in soils through different mechanisms, such as metal immobilization in the soil, improve soil fertility and enzyme activity (Al-Wabel et al., 2015). Soil enzymes are involved in nutrient cycling and availability to plants, and the enzymes activity can be used as an indicator of soil health (Zeng et al., 2007).

Biochar is a carbon-rich by-product resulting from the pyrolysis of biological residues in an oxygen-free environment (Meng et al., 2013; Ahmad et al., 2014). Biochar is characterized by an alkaline pH, large surface area, high porosity, high stability, and high amounts of oxygen-containing functional groups on its surface. Biochar contains valuable macro- and micro-nutrients (Lebrun et al., 2021; Liu et al., 2017). It has been used to absorb and immobilize trace element (TE) contaminants, including Pb, and alleviate the phytotoxicity in TE-contaminated soils (Ippolito et al., 2012; Li et al., 2017; Luo et al., 2014; Zwieten et al., 2010). Biochar influences heavy metals speciation and reduces metal phytoavailability through precipitation, sorption, ion exchange reactions, and alteration of soil microbial activity and community structure (Hammer et al., 2014; Liu et al., 2018). Bian et al. (2014) reported that wheat straw biochar significantly reduced Pb mobility and uptake by rice plants. Gao et al. (2020a) found that biochar transformed the heavy metals in soil from available speciation to stable speciation. Biochar may improve soil fertility and plant growth due to its prominent properties of water holding capacity, nutrition retention and soil enzyme activity (Hossain et al., 2010; Spokas et al., 2012).

Biochar has a limited immobilization capacity for heavy metals and cannot release an adequate amount of nutrients to effect soil fertility and plant growth (Gao et al., 2020b; Lehmann, 2007). Farm manure is an organic material that has attracted attention recently for its higher levels of fertility (Liang et al., 2017; Wang et al., 2020). It is a stabilized soil amendment that can enhance and restore soil organic matter, improve water retention and soil structure, and provide available nutrients, including nitrogen (N), phosphorus (P), potassium (K), calcium (Ca), magnesium (Mg), sulphur (S) and essential TE to promote plant growth (Gajalakshmi & Abbasi, 2008; Kumpiene, Lagerkvist & Maurice, 2008; Lannan, Erich & Ohno, 2013). Applying farm manure can also decrease the availability of heavy metals in soils and plants (Pichtel & Bradway, 2008). Numerous studies have shown that farm manure may reduce the exchangeable fraction of Pb in soil due to Pb ions’ strong affinity for organic complexation sites. Farm manure may reduce Pb phytoavailability by decreasing the soil bulk density to dilute Pb concentration, improve plants’ nutrient uptake, and form immobilized complex between humic acids and Pb (Caballero et al., 2009; Fleming et al., 2013; Kumpiene, Lagerkvist & Maurice, 2008).

Some studies have reported on the additive effects of biochar and other organic amendments to improve soil quality and plant performance in degraded or contaminated soil (Kammann, Glaser & Schmidt, 2016; Głąb et al., 2018). Biochar and farm manure retain nutrition and may stabilize inorganic contaminants through adsorption, binding, and co-precipitation (Karami et al., 2011; Kumpiene, Lagerkvist & Maurice, 2008; Cui et al., 2020; Wang et al., 2020). Chicken excrement is widely used as farm manure; however, there are few studies that compare biochar, chicken manure and their combined effects as a soil amendment in Pb contaminated soil. In this study, a pot culture experiment was conducted to investigate the effects of biochar and chicken manure, individually or in combination, on maize growth, leaves’ antioxidant enzyme activity, Pb uptake, and soil enzyme activities in 800 g·kg^−1^ Pb contaminated soil. The objective of the present study was to evaluate the additive effects of biochar in combination with chicken manure on maize growth and alleviating Pb stress.

## Materials and Methods

### Soil sampling and soil amendment materials

We sampled the surface soil at a depth of 0–20 cm from an agricultural field at the Henan University of Science and Technology, Luoyang, China. Soil samples were air-dried and crushed to pass through a 2-mm mesh. The soil was classified as Aquic Ustochrept (US soil taxonomy), and the soil texture was loamy (sand 48.95%, silt 32.24%, clay 18.81%). The soil had a pH of 7.6, 12.62 g·kg^−1^ organic matter, 0.56 g·kg^−1^ total N, 46.66 mg·kg^−1^ available N, 16.13 mg·kg^−1^ available P, 106.52 mg·kg^−1^ available K, and 10.23 mg·kg^−1^ total Pb.

Biochar was provided by the Sanli New Energy Company, Henan Province, China. It was derived from wheat straw pyrolyzed at 450 °C in an oxygen-free environment for 2 h. We analyzed the biochar’s properties according to Lu (2000)’s methodology. Basic biochar’s properties were: 46.8% organic C content, 5.9 g·kg^−1^ total N, 23.2 g·kg^−1^ K, 0.89 g·kg^−1^ available P, 0.53 g·kg^−1^ dissolved organic carbon, pH of 10.4, and 8.92 m^2^·g^−1^ surface area.

Dried, decomposed chicken manure was provided by Luoyang Qihe Ecological Agriculture Science and Technology Co. Ltd, Henan Province, China. Chicken manure’s properties were: pH of 6.7, 41.6% organic C content, 24.3 g·kg^−1^ total N, 34.3g·kg^−1^ K, 20.5 g·kg^−1^ available P, and 0.53 g·kg^−1^ dissolved organic carbon (DOC).

### Pot experiment

The pot experiment included six treatments: the control (CK, no amendment), low chicken manure (LM, chicken manure in soil at 20 g·kg^−1^), high chicken manure (HM, chicken manure in soil at 40 g·kg^−1^), low biochar (LB, biochar in soil at 20 g·kg^−1^), high biochar (HB, biochar in soil at 40 g·kg^−1^), and a combination of chicken manure and biochar (BM, biochar and chicken manure in soil each at 20 g·kg^−1^). Each plastic pot (20 cm diameter × 25 cm depth) contained 4 kg of sampling soil. All the amendments were thoroughly mixed into the soil. Pb was added to a level of 800 mg·kg^−1^ from a Pb (CH_3_COO)_2_ solution to simulate moderately Pb contaminated soil. To achieve an equilibrium condition, the simulated contaminated soil was incubated at room temperature for 2 months and irrigated with deionized water to maintain the water holding capacity at about 60%.

Three maize (*Zea mays* L. cv. Keda No. 16) seeds per plot were sown on May 10, 2019. At 10 days after germination, seedlings were hand-thinned to one plant per plot. There were no additional fertilizers were applied and watered regularly to maintain a soil moisture content of 60–70% of water holding capacity during the growth period. The pots were randomly arranged in the greenhouse, having eight replications of each treatment. Four replicates of each treatment were selected to measure the antioxidant enzyme activities in the leaves 45 days after sowing. At 80 days after sowing, the other four replicates were used to measure maize height, biomass, and Pb content in maize tissues. Soil samples were collected for analysis of soil available Pb, pH, and soil enzyme activity.

### Assays of antioxidant enzymatic activities in leaves

Leaf samples (0.5 g) from plants of each treatment group were collected and homogenized in 8 mL of 50 mM potassium phosphate buffer (pH 7.8) under ice-cold conditions to measure MDA content (Salah et al., 2015). MDA was tested using the thiobarbituric acid (TBA) reaction (Heath & Packer, 1968). Superoxide dismutase (SOD) activity levels were determined based on their ability to inhibit nitroblue tetrazolium (NBT) reduction by O^−2^ radicals (Madhava Rao & Sresty, 2000). Peroxidase (POD) activity levels were assessed using guaiacol as the substrate in 3 mL total volume (Ghosh & Singh, 2005). Catalase (CAT) activity levels were determined using the ultraviolet absorption method according to the H_2_O_2_ consumption rate at 240 nm by (Cakmak & Marschner, 1992).

### Plant analysis methods

Plant height was recorded with a ruler before harvesting. After plant harvest, roots, shoots, and leaves were separated, and were carefully washed with tap water followed by distilled water. The samples were put in an electric oven until a constant weight to record the dried roots, stems, and leaves biomass and stored for further analysis. The dried plant samples were ground to <0.25 mm in a Retsch MM 400 ball-mill and acid-digested to determine plant Pb concentration by inductively coupled plasma atomic emission spectrometry (ICP-AES, Varian AA240).

### Soil analysis methods

After harvesting, maize roots were shaken by hand to remove the loosely adhered soil to allow for the collection of rhizosphere soil. The rhizosphere soil samples were dried, ground, and sieved (2-mm sieve) to test for soil physicochemical parameters, diethylene triaminepentaacetic acid (DTPA) extractable Pb, and soil enzymes activity. Soil pH was measured using an acidity agent (soil water ration of 1: 5) (PHS-3C pH acidometer, China). Soil organic carbon was determined using the potassium dichromate oxidation method after digestion with concentrated sulfuric acid (Kalembasa & Jenkinson, 1973). The available N content in soil was alkali dispersed by 1 mol l^−1^ NaOH. The available P content was measured with by 0.5 mol l^−1^ NaHCO_3_ followed by the molybdenum blue colorimetry method using a UV-2300 spectrophotometer (Tianmei Technology Company, Jiaxing City, China). The available K content was determined using a flame photometer (M410, Sherwood, England). To determine the diethylene triaminepentaacetic acid (DTPA) extractable Pb concentration, we extracted 20 g dried soil with 50 mL of DTPA-TEA (triethanolamine) solution that had a pH of 7.3. The suspension was extracted and the Pb content was measured using inductively coupled plasma atomic emission spectrometry (ICP-AES, Varian AA240) (Lindsay & Norvell, 1978). Urease and sucrase activity were measured using a Perkin-Elmer Lambda 25 spectrophotometer (MA, USA) at a wavelength (λ) of 578 nm and 508 nm, respectively. Catalase activity was determined by hydrogen peroxide decomposition using potassium permanganate (Borowik, Wyszkowska & Wyszkowski, 2017).

### Calculating of bioconcentration factor and transfer factor

Two indices were used to evaluate an individual plant’s ability to accumulate and translocate Pb. The bioconcentration factor (BCF) was calculated as: *BCF* = *C*_*aboveground*_/*C*_*soil*_, where *C*_*aboveground*_ is the average concentration of Pb in stem and leaf tissues and *C*_*soil*_ is the Pb concentration in the soil. The transfer Factor (TF) was calculated using the equation: *TF* = *C*_*aboveground*_/*C*_*root*_, where *C*_*aboveground*_ is the average concentration of Pb in stem and leaf tissues and *C*_*root*_ is the Pb concentration in the roots (Cakmak & Marschner, 1992).

### Statistical analysis

Statistical analyses were conducted with one-way ANOVA using the least significant difference (LSD) test to determine whether the means were significantly different, *P* < 0.05 was considered to be significant. All experimental data were analyzed using SPSS software (ver. 22.0, SPSS Inc., Chicago, IL, USA), and all bar graphs were drawn using the Origin software (ver. 8.0).

## Results

### Maize plant height and dry weight

**Figure 1:**
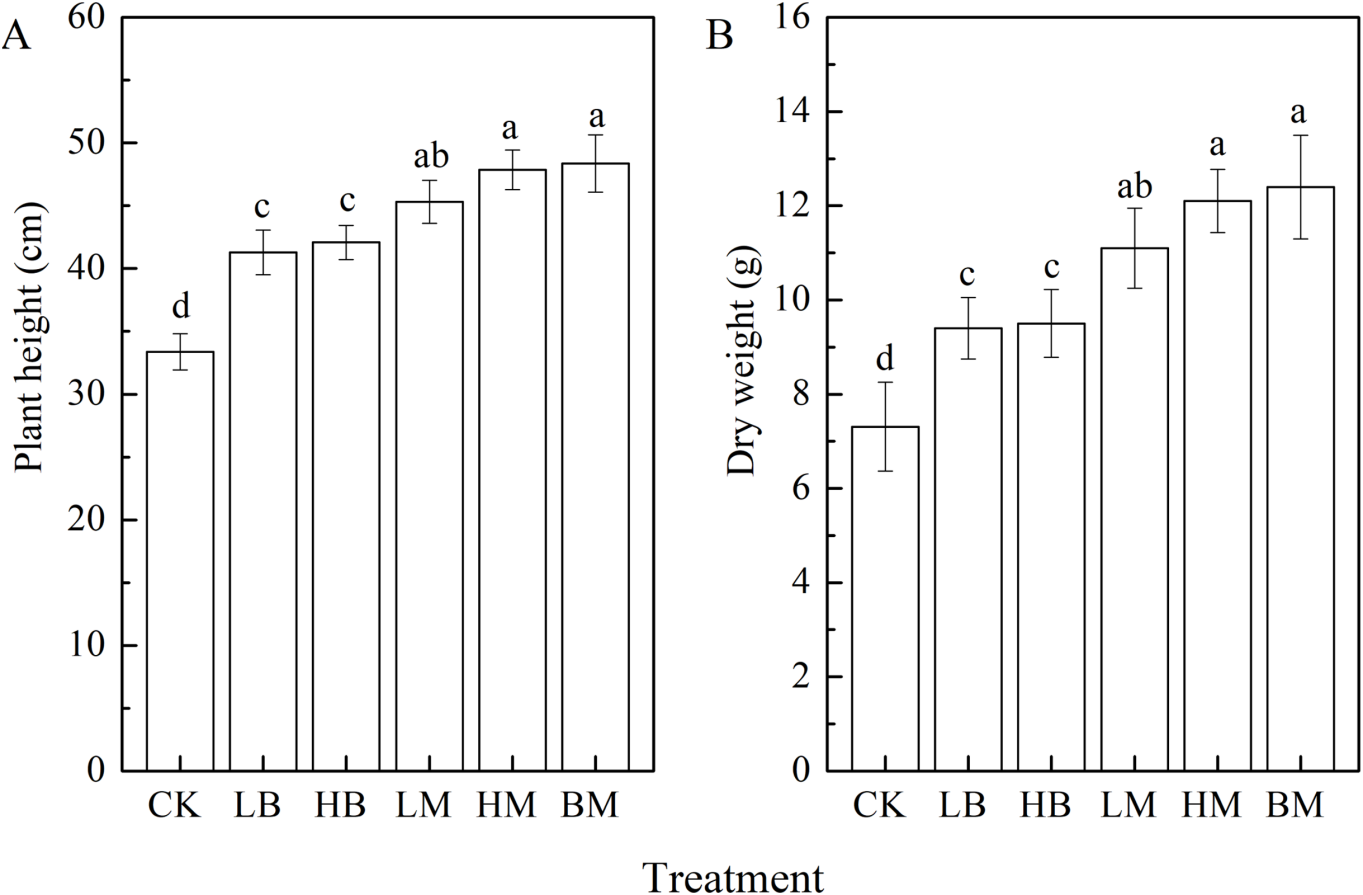
Plant height (A) and dry weight (B) of maize grown at Pb contaminated soil. CK, LB, HB, LM, HM, BM represent the control, biochar application of 20 g·kg^−1^ soil, biochar application of 40 g·kg^−1^ soil, chicken manure application of 20 g·kg^−1^ soil, chicken manure application of 40 g·kg^−1^ soil, a combination of biochar and chicken manure (each at 20 g·kg^−1^ soil), respectively. Values are means (±SD) of four replicates; different lowercase letters indicate significant differences among different soil amendment treatments according to LSD at *P* < 0.05.

Plant height and dry weight reflected the growth difference between the control and the five amended contaminated soils (Fig. 1). The control treatment, without biochar or chicken manure application, showed the shortest plant height and smallest dry weight. All five biochar and chicken manure treatments significantly improved maize height and dry weight (*P* < 0.05) compared to the control. Furthermore, chicken manure had a greater effect than biochar, and biochar together with chicken manure had the strongest effect of all. Compared to the control, LB, HB, LM, HM, and BM improved the plant height by 23.67%, 26.06%, 35.80%, 43.23%, and 44.894, respectively (Fig. 1A). They improved the dry weight by 28.59%, 29.96%, 51.84%, 65.53%, and 69.63%, respectively (Fig. 1B).

### MDA contents and antioxidant enzymatic activities

**Figure 2:**
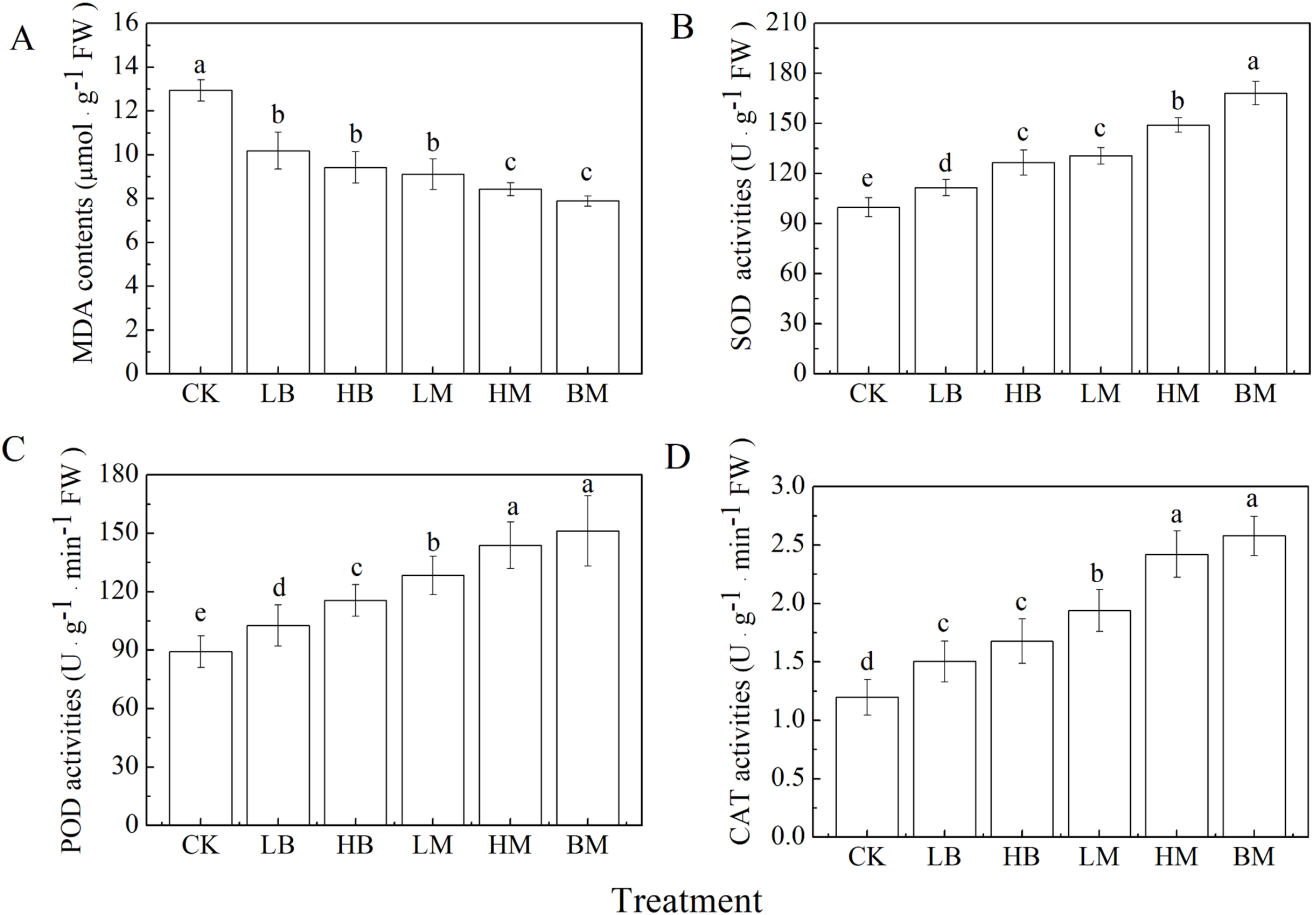
Malondialdehyde (MDA) contents (A) and activities of superoxide dismutase (SOD) (B), peroxidases (POD) (C), catalase (CAT) (D) in maize leaves grown at Pb contaminated soil. CK, LB, HB, LM, HM, BM represent the control, biochar application of 20 g·kg^−1^ soil, biochar application of 40 g·kg^−1^ soil, chicken manure application of 20 g·kg^−1^ soil, chicken manure application of 40 g·kg^−1^ soil, a combination of biochar and chicken manure (each at 20 g·kg^−1^ soil), respectively. Values are means (±SD) of four replicates; different lowercase letters indicate significant differences among different soil amendment treatments according to LSD at *P* < 0.05

All five biochar and chicken manure treatments significantly decreased MDA contents and increased antioxidant enzymatic activities as compared to the control (Fig. 2) (*P* < 0.05). The lowest MDA content was observed in the BM treatment, but there was no significant difference with the HM treatment (*P* > 0.05). No significant differences were found between the LB, HB, and LM treatments (*P* > 0.05).

The BM treatment demonstrated the most significant (*P* < 0.05) increase in SOD, POD, and CAT activities by 68.44%, 69.41%, and 115.27%, respectively, when compared to the control. Application of biochar and chicken manure at higher rates significantly (*P* < 0.05) increased SOD, POD, and CAT activities compared to the lower amendment application rate (Fig. 2). The exception was the biochar only amendment on CAT activities. Furthermore, chicken manure had a greater effect on antioxidant enzymatic activity than biochar.

### Pb concentration in maize

**Figure 3:**
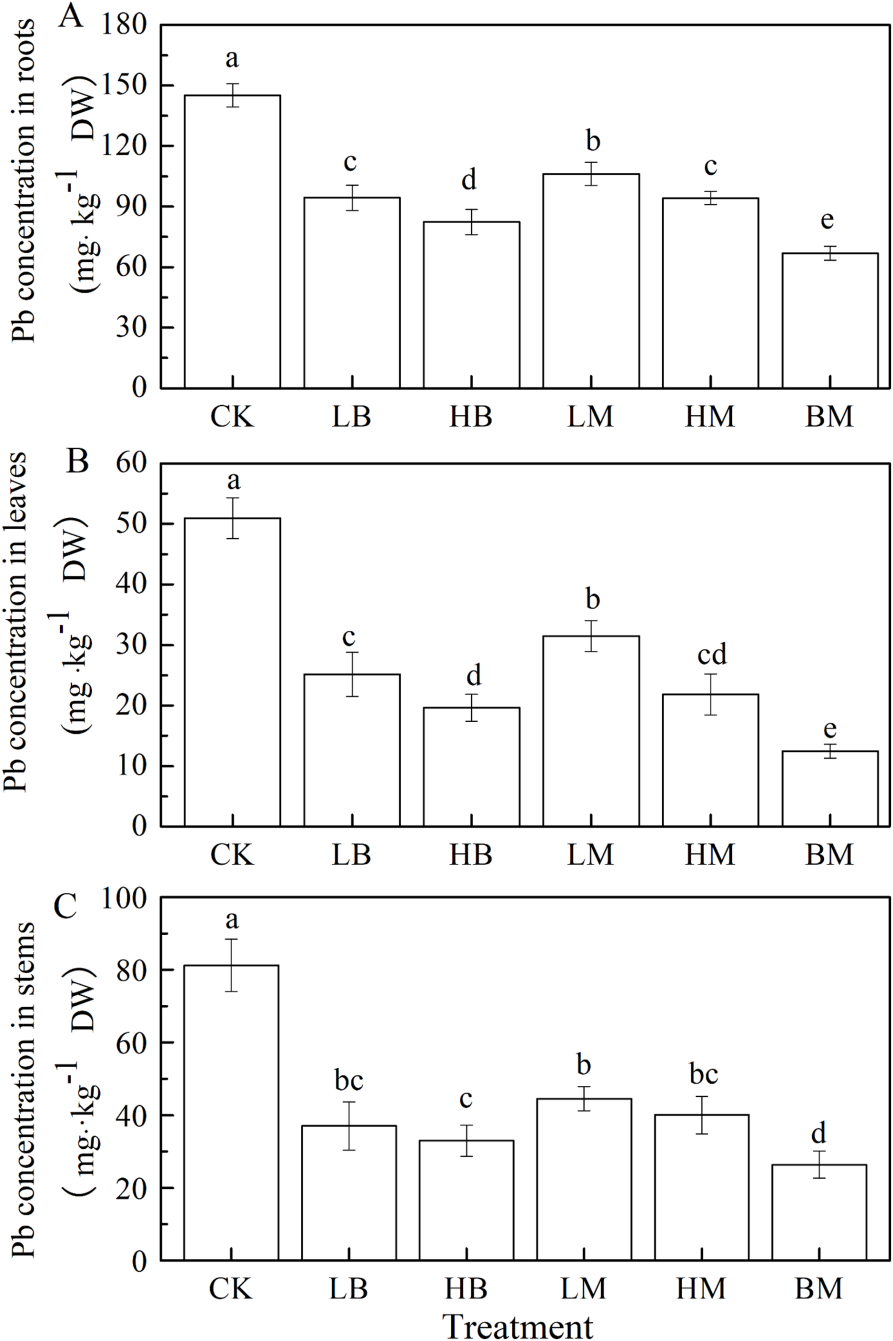
Pb concentrations in roots (A), leaves (B) and stems (C) of maize grown at Pb contaminated soil. CK, LB, HB, LM, HM, BM represent the control, biochar application of 20 g·kg^−1^ soil, biochar application of 40 g·kg^−1^ soil, chicken manure application of 20 g·kg^−1^ soil, chicken manure application of 40 g·kg^−1^ soil, a combination of biochar and chicken manure (each at 20 g·kg^−1^ soil), respectively. Values are means (±SD) of four replicates; different lowercase letters indicate significant differences among different soil amendment treatments according to LSD at *P* < 0.05.

Pb concentrations in maize tissue trended as root>stem>leave (Fig. 3). Amendments of LB, HB, LM, HM, and BM all significantly (*P* < 0.05) decreased the Pb concentrations in maize tissues. The combined biochar and chicken manure treatment was most effective in decreasing Pb concentration in maize roots, stems and leaves with 53.93%, 75.53% and 67.54%, respectively. The higher application rate of biochar and chicken manure treatments significantly decreased (*P* < 0.05) the Pb concentration in roots and leaves, compared to the lower application rate. However, no significant difference was found in the stems (*P* > 0.05). The HB treatment decreased the Pb concentration of the roots, stems and leaves by 43.24%, 61.45%, and 59.39%, respectively. The HM treatment also decreased the Pb concentration of the roots, stems, and leaves by 35.06%, 57.15%, and 50.74%, respectively.

### Effects of different soil amendments on soil physicochemical properties and available Pb content

Applying of biochar and chicken manure amendments alone or in combination all significantly (*P* < 0.05) decreased the soil DTPA extractable Pb concentrations (Table 1). The trend of DTPA extractable Pb concentrations in the soil was BM<HB<HM<LB<LM<CK (Table 1), and there were significant differences among the different treatments (*P* < 0.05). These results showed that biochar had a greater effect than chicken manure on decreasing the soil available Pb. The higher application rate of biochar and chicken manure treatments significantly decreased (*P* < 0.05) the soil DTPA extractable Pb concentration. Biochar and chicken manure in combination had the greatest impact on the reduction in Pb bioavailability.

**Table 1:** Effect of different treatments on soil physicochemical properties of Pb contaminated soil.

Treatments	pH	Soil organic carbon (g·kg^−1^)	Available N (mg·kg^−1^)	Available P (mg·kg^−1^)	Available K (mg·kg^−1^)	Available Pb (mg·kg^−1^)
CK	7.79 ± 0.09c	11.34 ± 0.22e	61.51 ± 1.49e	10.23 ± 0.78d	106.24 ± 4.23e	133.21 ± 11.63a
LB	8.21 ± 0.13b	12.28 ± 0.16d	73.47 ± 1.78d	13.85 ± 0.67c	115.07 ± 4.35d	103.52 ± 6.24c
HB	8.45 ± 0.14a	15.23 ± 0.21c	79.01 ± 1.88c	17.45 ± 1.21b	131.48 ± 5.12b	80.56 ± 4.30e
LM	7.57 ± 0.16d	16.37 ± 0.25c	82.01± 0.98b	15.45 ± 0.91c	121.28 ± 4.35c	115.32 ± 7.25b
HM	7.51 ± 0.09d	18.56 ± 0.34b	92.34 ± 4.12a	19.57 ± 1.12a	145.23 ± 5.07a	93.65 ± 4.14d
BM	7.91 ± 0.14c	19.23 ± 0.27a	91.23 ± 4.78a	19.43 ± 1.03a	143.46 ± 4.67a	64.35 ± 3.22f

Soil pH significantly (*P* < 0.05) increased due to amendments with HB and LB, but decreased with the application of HM and LM. No significant difference was found between BM and CK treatment (Table 1). As compared to the lower rate addition of biochar or chicken manure, higher rate of biochar significantly (*P* < 0.05) increased soil pH, while no significant decreased (*P* > 0.05) with higher rate of chicken manure. The application with higher rate of biochar showed the highest increase in soil pH by 8.4% compared to the control. All five biochar and chicken manure treatments significantly (*P* < 0.05) increased soil organic carbon, available nitrogen, available P, and available K contents compared to the control (Table 1). Furthermore, the HB and HM treatments increased the soil organic carbon, available nitrogen, available P, and available K content more than the LB and LM treatments.

### Effects of different soil amendments on soil enzyme activities

**Figure 4:**
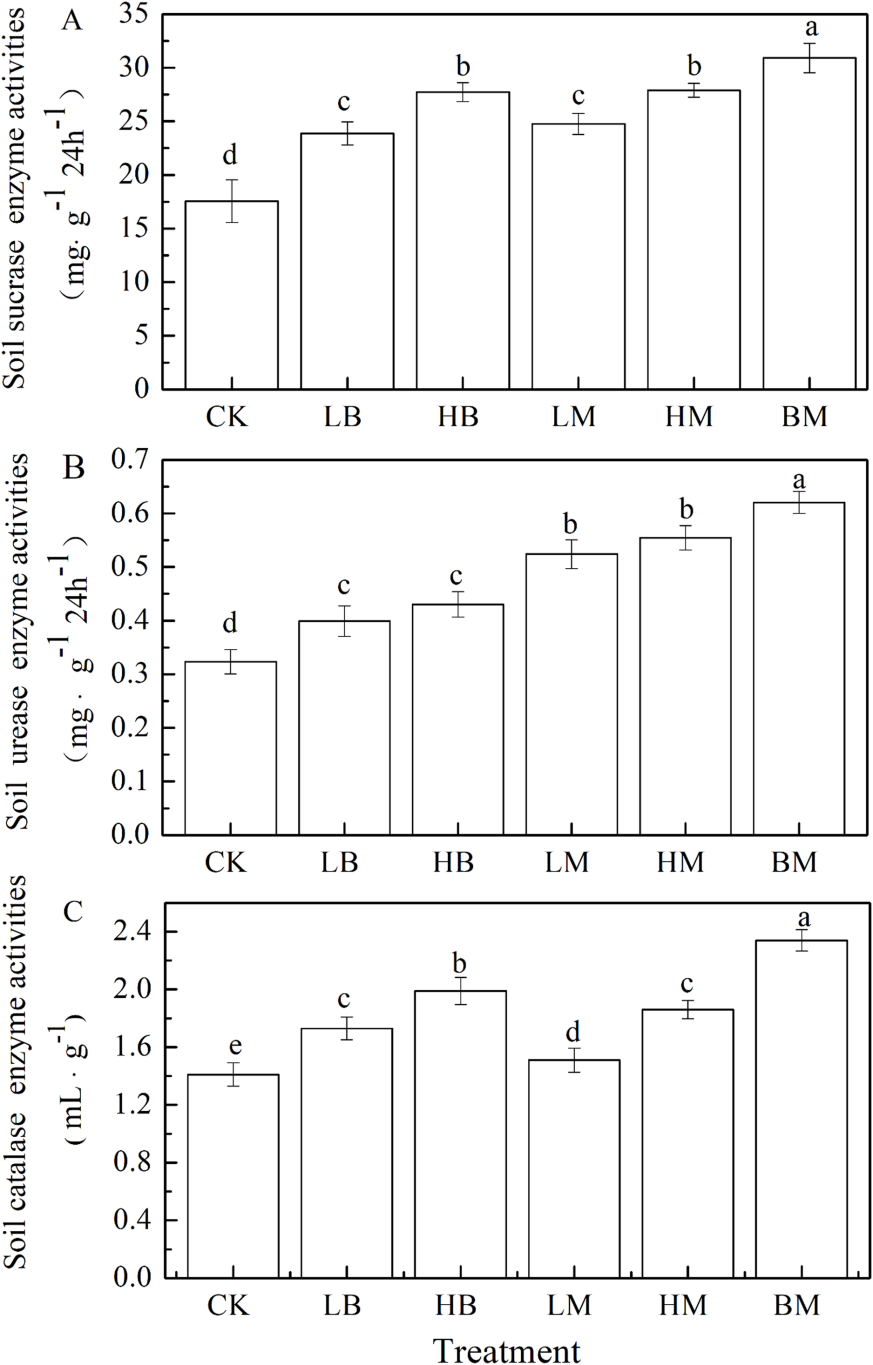
Soil enzyme activities of sucrase (A), urease (B) and catalase (C) of maize grown at Pb contaminated soil. CK, LB, HB, LM, HM, BM represent the control, biochar application of 20 g·kg^−1^ soil, biochar application of 40 g·kg^−1^ soil, chicken manure application of 20 g·kg^−1^ soil, chicken manure application of 40 g·kg^−1^ soil, a combination of biochar and chicken manure (each at 20 g·kg^−1^ soil), respectively. Values are means (±SD) of four replicates; different lowercase letters indicate significant differences among different soil amendment treatments according to LSD at *P* < 0.05.

Soil amended with LB, HB, LM, HM, and BM significantly increased sucrase, urease, and catalase activities by 34.21%, 53.02%, 40.95%, 58.81%, and 75.91% (Fig. 4A), 23.33%, 33.03%, 51.03%, 66.35%, and 81.82% (Fig. 4B) and 21.42%, 41.13%, 15.17%, 36.57%, and 65.75% (Fig. 4C), respectively, compared to the control. Biochar amendments were more effecting in catalase activity while chicken manure amendments had a greater impact on the urease activity. There were no significant (*P* > 0.05) differences on sucrase activity between the biochar and chicken manure amendments. Biochar and chicken manure in combination showed the largest increase in sucrase, urease and catalase activities in Pb-contaminated soil.

### Effects of different soil amendments on BCF and TF of Pb in maize

**Figure 5:**
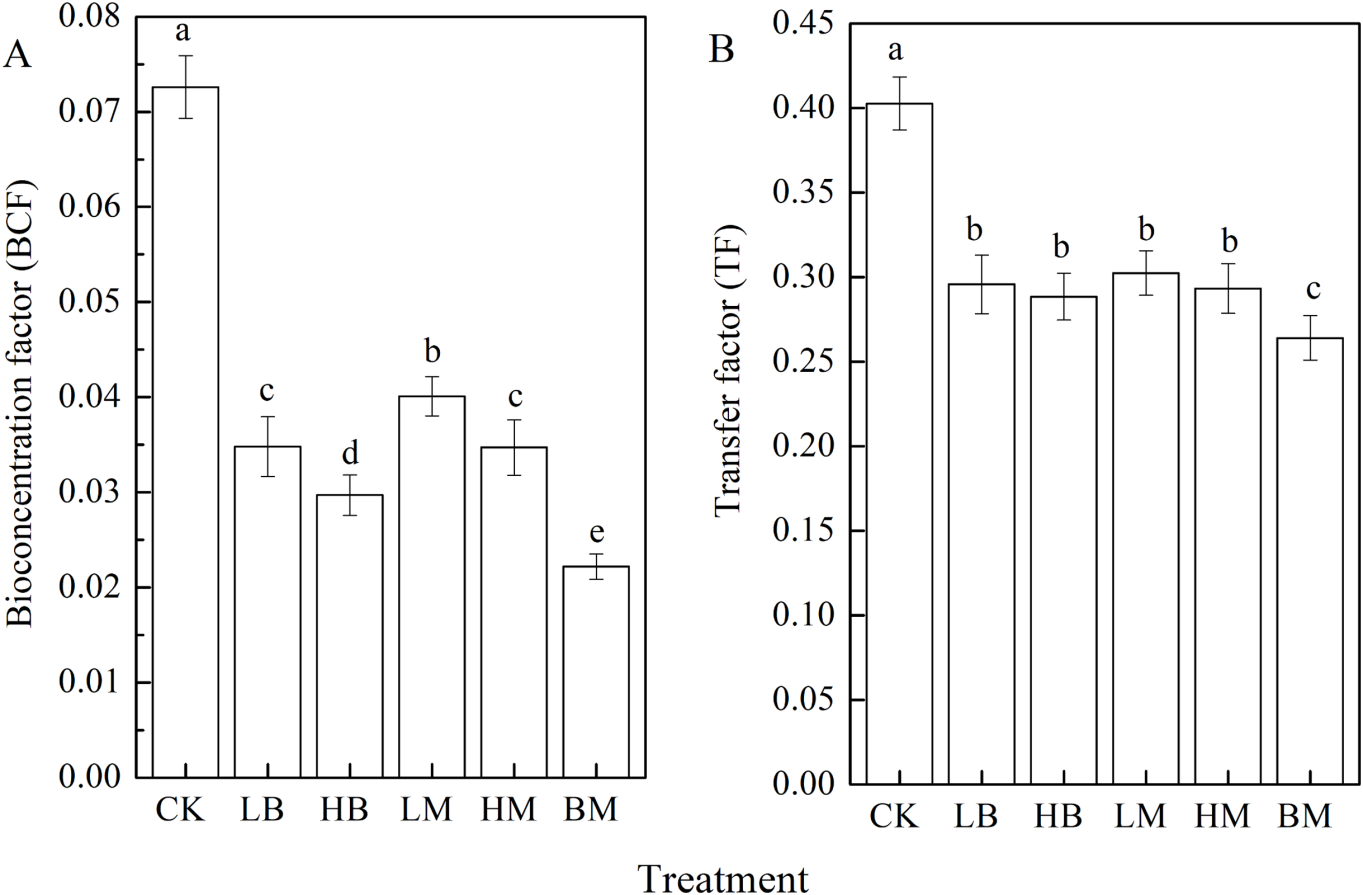
Bioconcentration factors (BCF) (A) and transfer factors (TF) (B) of maize grown at Pb contaminated soil. CK, LB, HB, LM, HM, BM represent the control, biochar application of 20 g·kg^−1^ soil, biochar application of 40 g·kg^−1^ soil, chicken manure application of 20 g·kg^−1^ soil, chicken manure application of 40 g·kg^−1^ soil, a combination of biochar and chicken manure (each at 20 g·kg^−1^ soil), respectively. Values are means (±SD) of four replicates; different lowercase letters indicate significant differences among different soil amendment treatments according to LSD at *P* < 0.05.

The five soil amendments significantly (*P* < 0.05) decreased Pb’s BCF and TF in maize (Fig. 5). Adding LB, HB, LM, HM, and BM decreased the BCF by 52.0%, 59.09%, 44.76%, 52.20%, and 69.42%, respectively, compared to the control. The most significant (*P* < 0.05) decrease in TF was observed in BM. In BM, TF decreased by 34.42% compared to the control, but no difference was found between LM, HM, LB, and HB (Fig. 5). These results showed that biochar and chicken manure in combination had the best effect, and that biochar was more effective than chicken manure in decreasing Pb translocation from the soil to maize.

## Discussion

The present study evaluated the effectiveness of applying biochar and/or chicken manure to improving physicochemical properties of soil, promoting plant growth, and reducing Pb uptake. The results showed that all of the biochar and chicken manure amendments improved the maize height and dry weight under Pb stress (Fig. 1). These results are congruent with previous studies which found that organic amendments increased plant growth and biomass under heavy metal stress (Baker, White & Pierzynski, 2011; Khan et al., 2013; Kiran & Prasad, 2019; Liu et al., 2018; Zhou et al., 2005). The chicken manure amendment alone was more effective at facilitating maize growth and increasing antioxidant enzyme activities in leaves compared to the addition of only biochar. Chicken manure was better able to provide essential nutrients directly to maize, improving the growth of the plant.

Soil organic carbon, N, P, and K are the major limiting factors for crop growth (Kumar, Swaminathan & Kumar, 2009). In this study, all soil amendments significantly increased soil organic carbon, available N, available P, and available K contents while decreasing the available Pb content in the soil as compared to the control (Table 1). Biochar contains numerous carbons, valuable macro-nutrients (especially N, P and K), and some metal ions (e.g., Ca^2+^ and Mg ^2+^) (Oustriere et al., 2016) which provide nutrients for plant growth. The surface charge and functional groups of biochar are conducive to the retention of soil nutrients. Biochar has a porous structure, large surface area, and high surface charge density, all of which are favorable to accumulating soil moisture, increasing soil porosity, reducing bulk density, and providing beneficial environment for plant growth. Chicken manure also contains numerous nutrients, which may increase soil nutrient content, improve the structure and stability of soil aggregates, and enhance soil microbial activity (Fleming et al., 2013).

The results showed that maize leaves’ SOD, POD, and CAT activities increased with the application of biochar and chicken manure, alone or in combination (Fig. 2). These amendments scavenged reactive oxygen species (ROS) and alleviated the oxidative damage to biomolecules (Wang et al., 2016). Chicken manure alone was more effective at increasing antioxidant enzyme activities in leaves than the addition of only biochar. Biochar in combination with chicken manure had the greatest effect on increasing antioxidant enzyme activities in leaves. The application of biochar and chicken manure improved the antioxidant enzymatic activity of maize by improving the physiochemical properties of the soil and decreasing Pb uptake (Table 1) (Lu et al., 2014; Walker, Clemente & Bernal, 2004; Wang et al., 2013; Xu et al., 2018).

Soil enzymatic activity was the most important factor of soil fertility and soil amendments (Kong & Chu, 2018; Ouyang et al., 2014). Soil microbial communities secrete intracellular and extracellular enzymes that play a role in the biogeochemical cycle of nutrients in the soil and contribute to the fertility and health of the soil (Mierzwa-Hersztek, Gondek & Baran, 2016). The present experiment demonstrated that biochar and chicken manure amendments significantly increased soil sucrase, urease, and catalase activities under Pb stress (Fig. 4), which are in agreement with previous studies (Awasthi et al., 2017; Bandara et al., 2017; Meng et al., 2018). The combination of biochar and chicken manure application showed the topmost activities of sucrose, urease and catalase in the Pb-contaminated soil, compared to the control. Increased enzymatic activities in the soil may be due to the reduction in Pb stress on microbiota. This may have been achieved by binding Pb in soil and transforming Pb into unavailable speciation (Khan et al., 2020; Naeem et al., 2021). Biochar and chicken manure may improve soil nutrients, water retention capacity, and porosity to create a suitable environment for the soil microbiota. The presence of compounds in biochar and chicken manure, including free radicals, minerals, volatile organics, and labile substrates, also reshape the microbial community to positively influence the enzymatic activities in the soil (Foster et al., 2016; Ge et al., 2010; Mierzwa-Hersztek, Gondek & Baran, 2016; Paz-Ferreiro et al., 2012).

The results showed that applying biochar and/or chicken manure amendments decreased Pb uptake by maize (Figs. 3, 5). Combining biochar and chicken manure decreased Pb uptake by maize most significantly. The biochar amendment had a greater effect than chicken manure on lowering Pb concentrations in maize tissues. Al-Wabel et al. (2015) found that applying biochar decreased Pb concentrations in maize plants. Gul et al. (2016) reported that livestock manure decreased Pb, Zn, Cr, Cu, and Cd concentrations in the roots and shoots of maize. Pb reduction in maize after applying biochar may be due to biochar’s ability to absorb heavy metals. This is possible due to biochar’s large surface area, porous structure, and high surface charge density (Liu et al., 2018; Xu, Cao & Zhao, 2013). The biochar amendment was more effective at increasing soil pH compared to the control. Increasing the soil pH alters heavy metals to their less mobile ionic form and controls heavy metal’s bioavailability in the soil, reducing the heavy metal ionic translocation from soil to maize tissues (Liu et al., 2018; Nie et al., 2018; Wang et al., 2013). The effects of chicken manure amendments in decreasing Pb concentration in maize tissues may be related to the surface charge, metal-binding compounds, humic substances, all of which can produce the adsorption, complexation, precipitation reactions with heavy metals (Park et al., 2011; Wang et al., 2013; Wu et al., 2017).

In the study, the combination of biochar and chicken manure was most effecting in decreasing Pb uptake by maize. There was an additive interaction between biochar and chicken manure to alleviate Pb toxicity and improve maize growth under Pb stress. There were several possible mechanisms for the additive effects of the combined application of biochar and chicken manure. On one hand, the addition of chicken manure compensates for the lack of nutrients in biochar (Sorrenti & Toselli, 2016). Applying biochar increases nutrient retention and prolongs the release period of the nutrients in chicken manure, effectively improving the utilization of organic fertilizer (Foster et al., 2016; Jones et al., 2016; Rehman et al., 2018). A combination of biochar and chicken manure can enhance soil organic carbon, pH, physical adsorption, and surface precipitation ability, of which can sorb and immobilize heavy metals (Belyaeva & Haynes, 2012; Karer et al., 2015; Liang et al., 2017). On the other hand, there is a positive interaction between biochar and chicken manure resulting in crop benefits. Biochar has an impact on the process of manure humification (Zeng et al., 2015) and its surface can be oxidized by the humus and microorganisms found in organic manure (Jones et al., 2016; Wu et al., 2017). The humic matter and mineral oxides in the biochar and manure mixture could produce heavy metals complex (Meng et al., 2018; Oustriere et al., 2016; Zeng et al., 2015). Moreover, biochar can provide an extra source of energy, microporous space and act as a carrier for microorganisms in manure and soil, thus providing a favorable environment for microbial growth to immobilize heavy metal (Liu et al., 2018; Liu et al., 2020; Nawab et al., 2018; Rehman et al., 2016). In brief, biochar and chicken manure are involved in reducing Pb uptake and improving maize growth and there is an additive effect when they are combined. The application ratio of biochar and chicken manure and the mechanisms underlying the effects of the combination of biochar and chicken manure should be investigated in more detail.

## Conclusions

The application of biochar or chicken manure alone or in combination, all improved maize growth, antioxidant enzymatic activities and soil enzymatic activities under Pb stress. These soil additives decreased Pb concentrations in maize plants and available Pb concentrations in the soil. The addition of biochar alone was more effective at increasing soil pH, decreasing Pb translocation from soil to maize and contributing to Pb immobilization. Chicken manure alone had a greater effect in promoting maize growth and antioxidant enzymatic activities in leaves. Where biochar and manure were applied as solo amendment, higher application rate was more effective at improving maize growth, antioxidant enzymatic activities and decreasing Pb concentrations in maize tissues and soil. Generally, biochar showed an additive effect with chicken manure for plant growth, antioxidant enzymatic activities and Pb uptake under Pb stress. Higher soil pH, lower available Pb concentrations, and stronger soil enzymatic activities under biochar and chicken manure applications could serve as tactics to be more widely adopted to bind/immobilize Pb. These findings suggest that biochar in combination with chicken manure may be an effective method for remediating Pb-contaminated soil and to promote plant growth.

## Supplemental Information

10.7717/peerj.11754/supp-1 Supplemental Information 1 Plant height and dry weight of maize grown at lead (Pb) contaminated soil of 800 mg·kg^−1^. CK, LB, HB, LM, HM, BM represent the control, biochar application of 20 g·kg^−1^ soil, biochar application of 40 g·kg^−1^ soil, chicken manure application of 20 g·kg^−1^ soil, chicken manure application of 40 g·kg^−1^ soil, a combination of biochar and chicken manure (each at 20 g·kg^−1^ soil), respectively. Values are means (±SD) of four replicates; different lowercase letters indicate significant differences among different soil amendment treatments according to LSD at *P* < 0.05

10.7717/peerj.11754/supp-2 Supplemental Information 2 Malondialdehyde (MDA) contents and activities of superoxide dismutase (SOD), peroxidases (POD), catalase (CAT) in maize leaves. CK, LB, HB, LM, HM, BM represent the control, biochar application of 20 g·kg^−1^ soil, biochar application of 40 g·kg^−1^ soil, chicken manure application of 20 g·kg^−1^ soil, chicken manure application of 40 g·kg^−1^ soil, a combination of biochar and chicken manure (each at 20 g·kg^−1^ soil), respectively. Values are means (±SD) of four replicates; different lowercase letters indicate significant differences among different soil amendment treatments according to LSD at *P* < 0.05

10.7717/peerj.11754/supp-3 Supplemental Information 3 Pb concentrations in roots, leaves and stems of maize.

10.7717/peerj.11754/supp-4 Supplemental Information 4 Soil available Pb concentrations and pH values.

10.7717/peerj.11754/supp-5 Supplemental Information 5 Bioconcentration factors (BCF) and transfer factors (TF) of maize.

10.7717/peerj.11754/supp-6 Supplemental Information 6 Soil enzyme activities of sucrase, urease and catalase of maize.

## Additional Information and Declarations

### Data Availability

The following information was supplied regarding data availability: Raw measurements are available in the Supplemental Files.
